# Exploring the Bi-Directional Association between Tobacco and E-Cigarette Use among Youth in Canada

**DOI:** 10.3390/ijerph16214256

**Published:** 2019-11-01

**Authors:** Sarah Aleyan, Mahmood R. Gohari, Adam G. Cole, Scott T. Leatherdale

**Affiliations:** 1 School of Public Health and Health Systems, University of Waterloo, Waterloo, ON N2L 3G1, Canada; mgohari@uwaterloo.ca (M.R.G.); adam.cole@uoit.ca (A.G.C.); sleather@uwaterloo.ca (S.T.L.); 2 Department of Addictions, King’s College London, London SE5 8BB, UK; 3 Faculty of Health Sciences, Ontario Tech University, Oshawa, ON L1G 0C5, Canada

**Keywords:** bi-directional, e-cigarette, tobacco, electronic cigarettes, youth

## Abstract

Research has demonstrated associations between e-cigarette use and tobacco use among youth. However, few studies have examined whether reciprocal relationships exist between e-cigarette and tobacco use. The objective of this study was to examine whether bi-directional associations exist between e-cigarette and tobacco use in a large longitudinal sample of Canadian youth. A longitudinal sample of secondary students (n = 6729) attending 87 schools in Ontario and Alberta, Canada, who completed the COMPASS student questionnaire across three waves (from 2014–2015 to 2016–2017) was identified. Using cross-lagged models, we explored bi-directional associations between current tobacco and e-cigarette use, adjusting for relevant covariates. Our findings showed that current e-cigarette use predicted subsequent tobacco use between Wave 1 (W_1_) and Wave 2 (W_2_) of the study (W_1–2_: OR = 1.54, 95% CI = 1.37–1.74). Similarly, current tobacco use predicted e-cigarette use during earlier waves of the study (W_1–2_: OR = 1.43, 95% CI = 1.30–1.58). However, these relationships dissipated in later waves, when tobacco use no longer predicted e-cigarette use (W_2–3_: OR = 1.07, 95% CI = 0.99–1.16). This study extends prior work that focused mainly on the association between e-cigarette and subsequent tobacco use. Specifically, our findings portray a more complex relationship, where e-cigarette use may influence and be influenced by tobacco use.

## 1. Background

Adolescent use of electronic cigarettes (e-cigarettes) has been on the rise in recent years both globally and within Canada [1,2,3]. The prevalence of past 30-day e-cigarette use among Canadian youth aged 15–19 years rose from 2.6 to 6.3 % between 2013 and 2015 [4]. The emergence of these novel devices has sparked concerns within the public health community that e-cigarette use among adolescents may re-normalize smoking behaviors [5,6].

Several longitudinal studies within the U.S. have demonstrated that the baseline use of e-cigarette use among non-smoking youth is associated with subsequent smoking initiation [7,8,9,10]. The associations between e-cigarette use and subsequent smoking initiation have also been demonstrated in studies conducted in England, Scotland and Canada [11,12,13,14]. A recent meta-analysis that examined nine longitudinal studies comprised of adolescents and young adults demonstrated that ever-users of e-cigarettes were 3.63 times more likely to smoke cigarettes at follow-up, in comparison to never-users of e-cigarettes [15]. These findings remained significant even after adjusting for numerous known risk factors for smoking, such as sensation-seeking tendencies [15].

Despite evolving evidence supporting public health concerns that e-cigarette use is associated with smoking behaviors among adolescents, most studies to date have focused specifically on the uni-directional relationship between baseline use of e-cigarettes among non-smoking youth and subsequent smoking behaviors. To our knowledge, only two studies have examined whether bi-directional relationships exist between e-cigarette use and smoking (i.e., whether smoking behaviors at baseline predict subsequent e-cigarette use and vice versa). The first longitudinal study conducted among a sample of 808 high school students in Connecticut identified a uni-directional relationship, according to which past-month use of e-cigarettes was associated with future cigarette smoking across three waves, but past-month cigarettes smoking was not associated with subsequent use of e-cigarettes [16]. The second longitudinal study conducted among a sample of 1152 11–18-year-olds in Great Britain identified a bi-directional relationship between ever use of e-cigarettes and subsequent cigarette smoking initiation, and between ever having smoked cigarettes and subsequent e-cigarette use [17]. In conjunction with the Addictions Hypothesis [18], exposure to nicotine during adolescence may lead to an increased liability to experiment with other nicotine products; this may explain the bi-directional relationships observed in previous work. To date, no studies have explored the potential bi-directional relationship between tobacco use and e-cigarette use in Canada where there are key distinctions in regulations surrounding the sale of e-cigarettes. In contrast to the U.S. and the U.K., where electronic cigarettes are widely available and nicotine-containing e-cigarettes are more accessible, the more restrictive regulatory environment present within Canada during the time of the study may have implications with respect to associations observed between e-cigarette and tobacco use. As such, the objective of this study was to examine the bi-directional association between tobacco use and e-cigarette use in a large longitudinal sample of Canadian youth. 

## 2. Methods

### 2.1. Design

The COMPASS study is a 9-year (2012–2021) prospective cohort study designed to collect longitudinal data from secondary students in grades 9–12 and the schools that they attend in Canada [19]. This study used longitudinal student-level data over three waves [Wave 1 (W_1_): 2014–2015; Wave 2 (W_2_): 2015–2016; Wave 3 (W_3_): 2016–2017] of the COMPASS study [19]. A more detailed description of the COMPASS study and its methods are available in print [19] or online (www.compass.uwaterloo.ca). The University of Waterloo Research Ethics Board along with participating school boards approved all procedures used, including the use of active-information passive-consent procedures.

### 2.2. Participants

In order to explore longitudinal changes among individuals, student-level data from W_1_ to W_3_ were linked over time. Qian and colleagues described the procedure and methods used to link student responses across multiple waves in more detail [20]. The main reasons for non-linkage included students dropping out of school, transferring to different schools, being on spare/absent during data collections, and the provision of inaccurate data for the linkage measures used. Overall, the linked sample used within this study consisted of 9th- and 10th-grade students at W_1_ who participated in all three waves of the COMPASS study (n = 6939 students). Students who had missing data for any covariates of interest (n = 210) were excluded, leaving a final analytic sample of 6729 students with three waves of data.

### 2.3. Outcome Measures 

Using a previously validated self-report measure [21], cigarette smoking behaviors were assessed at each wave by asking students to report whether they had smoked cigarettes within the last 30 days. Students were also asked to report whether they had used other tobacco products including cigarillos, little cigars, cigars, smokeless tobacco, and hookah within the past 30 days. Individuals who reported using cigarettes or other tobacco products at least once within the past 30 days were classified as current (past 30-day) tobacco users.

Current use of e-cigarettes was examined at each wave by asking the students to report whether they had used e-cigarettes within the last 30 days. Individuals who reported using e-cigarettes at least once within the last 30 days were classified as current (past 30-day) e-cigarette users.

### 2.4. Baseline Covariates

Students self-reported their demographic characteristics, including gender (male or female), grade (9, 10, 11, 12) and ethnicity (Black, White, Asian, Latin-American, Aboriginal, Other) at baseline. Since having friends that smoke, weekly spending money, current cannabis use, and binge drinking behaviors have been shown to be correlates of smoking behavior [22,23] , they were included in the model. Social influences for smoking were measured at each wave by asking “Your closest friends are the friends you like to spend the most time with. How many of your closest friends smoke cigarettes?” with response options of “None”, “1 friend”, “2 friends”, “3 friends”, “4 friends”, “5 friends or more”. Students’ weekly spending money was also measured at each wave by asking “About how much money do you usually get each week to spend on yourself or to save?” with response options of “zero”, “$1–$5”, “$6–$10”, “$11–$20”, “$21–$40”, “$41–$100”, “More than $100”, and “I don’t know how much money I get each week”. Current cannabis use was measured at each wave by asking “In the last 12 months, how often did you use marijuana or cannabis (a joint, pot, weed, hash)?” with response options ranging from “I have never used marijuana” to “every day”. Individuals who reported using cannabis at least once within the past month were classified as current (past 30-day) cannabis users; otherwise, they were categorized as non-current users. Current binge drinking was assessed at each wave by asking “In the last 12 months, how often did you have 5 or more drinks on one occasion?” with response options ranging from “I have never done this” to “Daily or almost daily”. Individuals who reported having 5 or more drinks at least once a month were classified as current (past 30-day) binge drinkers; otherwise, they were classified as non-current users.

## 3. Analyses

Descriptive statistics were used to examine the baseline characteristics of the linked longitudinal sample and the prevalence of current (past 30-day) e-cigarette use and tobacco use in each wave of the study. McNemar tests were used to examine whether there were significant changes in current e-cigarette use and tobacco use over time.

In order to answer our main research question, auto-regressive cross-lagged models simultaneously tested: (1) the auto-regressive effects of tobacco use and e-cigarette use at each wave (i.e., the stability of use of each product over time) and (2) the bi-directional relationships between e-cigarette use and tobacco use at each wave (i.e., whether e-cigarette use at W_1_ predicted subsequent tobacco use at W_2_ and vice versa). The fit of the cross-lagged models to our sample data was assessed using a range of fit indices, including the residual mean squared error (RMSE), the comparative factor index (CFI), and the Tucker–Lewis Index (TLI). The auto-regressive cross-lagged analysis was performed within a structural equational modelling (SEM) framework using robust mean and variance-adjusted weighted least-squares estimation (WLSMV) in the Mplus software program. Current (past 30-day) tobacco use and e-cigarette use were modelled as binary outcomes using the logit link function. Path estimates were then exponentiated in order to obtain odds ratios (ORs). The models adjusted for baseline characteristics, i.e., gender, grade, and ethnicity. The models also accounted for having friends that smoke, weekly spending money, current cannabis use, and current binge drinking at each wave. Our models also adjusted for the nested structure of the data (i.e., students nested within schools). 

## 4. Results 

Table 1 presents the baseline characteristics of the linked longitudinal sample (n = 6729). The gender distribution of the linked sample was approximately equal (52.2% female), and there were more 9th-grade than 10th-grade students (56.1% versus 43.9%). Furthermore, the majority of the study sample identified as White (76.1%). Table 2 presents the prevalence of current e-cigarette and tobacco use at each wave of the study. Unsurprisingly, our results demonstrated a significant increase in e-cigarette and tobacco use within the linked sample over time (*p* < 0.001).

**Figure 1:**
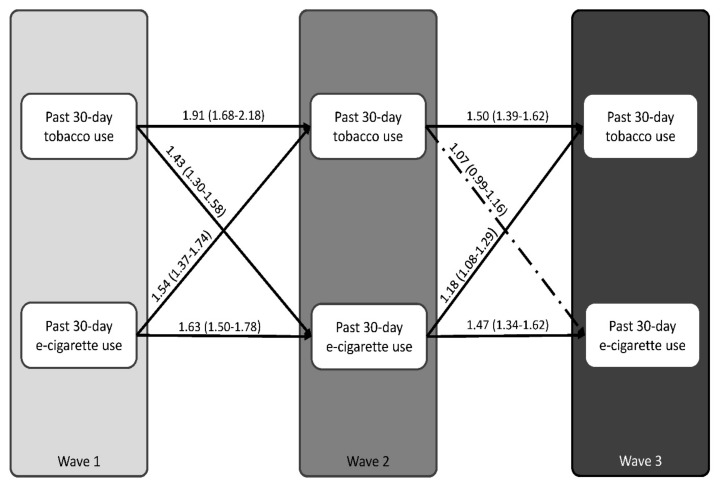
Relationships between current tobacco use and e-cigarette use among youth participating in all three waves of the study (n = 6729), adjusted for gender, grade, ethnicity, having friends who smoke, weekly spending money, cannabis use, and binge-drinking behaviors. Path estimates were exponentiated to obtain odds ratios (ORs). Adjusted ORs and 95% confidence intervals are presented for all pathways. The solid lines indicate significant effects (*p* < 0.05), while the dashed lines indicate non-significant effects (*p* > 0.05). Model fit indices: residual mean squared error (RMSE) = 0.032, comparative factor index (CFI) = 0.81, Tucker–Lewis Index (TLI) = 0.69.

Figure 1 presents the results of the auto-regressive cross-lagged models. The RMSE, CFI, and TLI indices showed adequate fit of the model to our sample data (RMSE = 0.032, CFI = 0.81, and TLI = 0.69). Controlling for relevant covariates, we identified significant auto-regressive effects for both current tobacco use and e-cigarette use at each wave; for example, current tobacco use at W_1_ predicted current tobacco use at W_2_ (OR = 1.91; 95% CI = 1.68–2.18). Similarly, current e-cigarette use at W_1_ predicted current e-cigarette use at W_2_ (OR= 1.63; 95% CI = 1.50–1.78). As shown in Figure 1, between W_1_ and W_2_, we identified a significant bi-directional association between current tobacco use and e-cigarette use: current tobacco users at W_1_ had 1.43 times higher odds of being current e-cigarette users at W_2_ (95% CI = 1.30–1.58). Similarly, current e-cigarette users at W_1_ had 1.54 times higher odds of being current tobacco users at W_2_ (95% CI = 1.37–1.74). As indicated in Figure 1, a significant uni-directional association was observed between current e-cigarette use and tobacco use between W_2_ and W_3_: current e-cigarette users at W_2_ had 1.18 times higher odds of being tobacco users at W_3_ (95% CI = 1.08–1.29).

## 5. Discussion

This is the first Canadian study to examine bi-directional associations between current e-cigarette use and tobacco use using longitudinal data from a large sample of youth. Consistent with previous evidence [15], our findings demonstrated that e-cigarette use was prospectively associated with subsequent tobacco use across all three waves of the study. Importantly, we found that this risk was bi-directional, such that current tobacco use was prospectively associated with subsequent e-cigarette use during earlier waves of the study. However, these bi-directional relationships appeared to dissipate in later waves, when tobacco use no longer predicted e-cigarette use. We also found that rates of past-month tobacco use and e-cigarette use increased across the three waves examined, demonstrating a rise in the number of students that were using tobacco products and e-cigarettes over time. These findings are consistent with those of national studies demonstrating higher rates of tobacco and e-cigarette use as youth progress to higher grades [4].

Several possible explanations may account for the bi-directional association between e-cigarette use and tobacco use observed in earlier waves of the study. It is possible that early exposure to nicotine during adolescence leads to an increased liability to experiment with other nicotine products. Previous research has shown that youth exhibit an enhanced sensitivity to the rewarding effects of nicotine, specifically during early adolescence [24,25]. It is important to note that though e-cigarettes containing nicotine were not approved for sale nationally during the time of this study, previous research has demonstrated that nicotine-containing e-cigarettes were widely available within the Canadian market [26]. It is also possible that there are common underlying factors that predispose youth to the use of both e-cigarettes and tobacco, as suggested by the Common Liability Theory [27]. For instance, youth who display rebelliousness and sensation-seeking characteristics may be more likely to experiment with both vaping and tobacco use (irrespective of the order of use). Future research should examine potential mediators (e.g., sensation seeking, rebelliousness) to gain a deeper understanding of the underlying mechanisms that account for the bi-directional relationships observed in earlier waves of the study.

Interestingly, these bi-directional associations were no longer apparent in later waves; for instance, past-month tobacco use behaviors no longer predicted subsequent e-cigarette use between waves 2 and 3 of the study. These differences may be attributable to changes in provincial legislation that prohibited the sale of e-cigarettes to those under 19 years in Ontario beginning January 2016 (i.e., in wave 2) (https://www.ontario.ca/page/electronic-cigarette-vape-rules#section-0). Prior to January 2016, no age restrictions were in place within Ontario relating to the sale of e-cigarettes. It is possible that this new policy may have reduced the accessibility of e-cigarettes to youth, thus resulting in the diminished effects seen here. While this requires further evaluation, previous research has demonstrated that interventions involving the disruption of tobacco sales to minors are associated with substantial declines in youth tobacco use [28]; the same may hold true with e-cigarettes. Another possible explanation is that the differences observed across waves are attributable to age-related effects. In other words, the role that e-cigarettes play in tobacco use patterns may be more prominent among younger versus older adolescents. These explanations are unlikely to be mutually exclusive, and future work should examine these potential influences to gain a better understanding of the underlying mechanisms that led to these observed effects.

Our study findings offer novel insight by illustrating the complexity underlying transitions in smoking behavior that resulted in the bi-directional associations observed. In contrast to the majority of research that has focused predominantly on the role that e-cigarettes play in smoking behavior [15], our findings offer a more comprehensive picture by exploring associations between tobacco use and subsequent e-cigarette use. Our findings suggest that nicotine delivery system specific intervention efforts targeting both tobacco use and e-cigarette use, may be warranted. 

Our findings hold important implications, given the recent changes in federal regulations introduced in May 2018 that now allow for the sale of nicotine-containing e-cigarettes in Canada. Under the new federal laws, the entry of large multi-national corporations that sell nicotine-containing e-cigarettes, including JUUL, may result in a shift within the Canadian market [29]. Our findings lend support to the need for policies that discourage youth from using e-cigarettes, particularly nicotine-containing e-cigarettes, as nicotine has been seen to affect the developing adolescent brain [24,30]. Furthermore, the Tobacco and Vaping Product Act (TVPA) now permit e-cigarette companies to advertise their products (with the exception of lifestyle advertisements and advertisements considered appealing to youth), with no restrictions regarding where these ads may appear [31]. Given the known associations between tobacco advertising and initiation of tobacco use among youth [32], regulations such as ad placement restrictions may act as a potential strategy to limit youth exposure to e-cigarette advertisements.

The study has several strengths including the use of a large longitudinal sample of youth from two Canadian jurisdictions, Ontario and Alberta, that were followed across three consecutive waves (2014–2017). The study also used a cross-lagged modelling approach that allowed for the examination of the stability and directionality of relationships between tobacco use and e-cigarette use over time. Inevitably, our study was also subject to various limitations. First, our measures of e-cigarette and tobacco use were limited to use within the past 30 days, which precluded characterizing different levels of consumption. Secondly, though specific characteristics of e-cigarettes may have been related to e-cigarette use and tobacco use over time, the current study did not have any measures that could be used to distinguish between the types of e-cigarettes being used (e.g., nicotine vs. non-nicotine, flavored/non-flavored). These product features should be considered in future work focused on examining associations between e-cigarette and tobacco use longitudinally. Thirdly, the presence of environmental factors that may have been related to e-cigarette and tobacco use (e.g., presence of provincial restrictions on youth access to e-cigarettes) were not examined in this study.

## 6. Conclusions 

This study extends previous work that focused predominantly on the association between e-cigarette use and tobacco use. Specifically, our findings demonstrate a more complex association, according to which e-cigarette use appears to influence and be influenced by tobacco use among youth. These findings underscore the need for comprehensive interventions aimed at preventing the use of both tobacco and e-cigarettes and indicate that such efforts need to be made early in the education cycle, prior to patterns of use becoming established. Given how rapidly the nicotine market has been evolving, continued monitoring and surveillance of patterns of e-cigarette and tobacco use are warranted.

## Figures and Tables

**Table 1:** Baseline characteristics of the linked longitudinal sample (n = 6729).

Student Characteristics			
		N	(%)
Gender	Female	3502	52.2%
	Male	3207	47.8%
Grade	9	3771	56.0%
	10	2958	44.0%
Ethnicity	White	5092	76.1%
	Black	210	3.1%
	Latin-American	125	1.9%
	Asian	361	5.4%
	Aboriginal	148	2.2%
	Other	759	11.3%
Weekly Spending Money	$0	1506	26.0%
	$1–20	2253	44.1%
	$20–100	1322	22.8%
	Over $100	412	7.1%
Current cannabis use	Yes	388	5.8%
	No	6264	94.2%
Current binge drinking	Yes	1999	29.7%
	No	4725	70.3%
Number of friends who smoke	None	5178	77.7%
	1–2	1155	17.3%
	3 or more	336	5.0%

**Table 2:** Prevalence of current (past 30-day) tobacco users and tobacco users at each wave of the study, 2014–2017 COMPASS study.

Outcome Measures	Linked Sample (Grade 9 and 10 at Baseline) (n = 6729)		
	Wave 1 n (%)	Wave 2 n (%)	Wave 3 n (%)
Tobacco users	263 (3.9%)	483 (7.2%)	722 (10.7%)
E-cigarette users	382 (5.7%)	476 (7.1%)	639 (9.5%)

## References

[1] Arrazola R.A., Singh T., Corey C.G., Husten C.G., Neff L.J., Apelberg B.J., Bunnell R.E., Choiniere C.J., King B.A., Cox S. (2015). Tobacco use among middle and high school students—United States, 2011–2014. MMWR. Morb. Mortal. Wkly. Rep..

[2] Montreuil A., MacDonald M., Asbridge M., Wild T.C., Hammond D., Manske S., Rutherford E. (2017). Prevalence and correlates of electronic cigarette use among Canadian students: Cross-sectional findings from the 2014/15 Canadian Student Tobacco, Alcohol and Drugs Survey. CMAJ Open.

[3] Gravely S., Fong G.T., Cummings K.M., Yan M., Quah A.C.K., Borland R., Yong H., Hitchman S.C., McNeill A., Hammond D. (2014). Awareness, trial, and current use of electronic cigarettes in 10 countries: Findings from the ITC project. Int. J. Environ. Res. Public Health.

[4] Reid J.L., Hammond D., Rynard V.L., Madill C.L., Burkhalter R. (2017). Tobacco Use in Canada: Patterns and Trends, 2017 Edition.

[5] Stanwick R. (2015). E-cigarettes: Are we renormalizing public smoking? Reversing five decades of tobacco control and revitalizing nicotine dependency in children and youth in Canada. Paediatr. Child Health.

[6] Stanbrook M.B. (2016). Electronic cigarettes and youth: A gateway that must be shut. CMAJ.

[7] Barrington-Trimis J.L., Urman R., Berhane K., Unger J.B., Cruz T.B., Pentz M.A., Samet J., Leventhal A.M., McConnell R. (2016). E-Cigarettes and Future Cigarette Use. Pediatrics.

[8] Miech R., Patrick M.E., O’Malley P.M., Johnston L.D. (2017). E-cigarette use as a predictor of cigarette smoking: Results from a 1-year follow-up of a national sample of 12th grade students. Tob. Control.

[9] Wills T.A., Knight R., Sargent J.D., Gibbons F.X., Pagano I., Williams R.J. (2018). Longitudinal study of e-cigarette use and onset of cigarette smoking among high school students in Hawaii. Tob. Control.

[10] Primack B.A., Soneji S., Stoolmiller M., Fine M.J., Sargent J.D. (2015). Progression to traditional cigarette smoking after electronic cigarette use among us adolescents and young adults. JAMA Pediatrics.

[11] Best C., Haseen F., Currie D., Ozakinci G., MacKintosh A.M., Stead M., Eadie D., MacGregor A., Pearce J., Amos A. (2017). Relationship between trying an electronic cigarette and subsequent cigarette experimentation in Scottish adolescents: A cohort study. Tob. Control.

[12] Aleyan S., Cole A., Qian W., Leatherdale S.T. (2018). Risky business: A longitudinal study examining cigarette smoking initiation among susceptible and non-susceptible e-cigarette users in Canada. BMJ Open.

[13] Conner M., Grogan S., Simms-Ellis R., Flett K., Sykes-Muskett B., Cowap L., Lawton R., Armitage C.J., Meads D., Torgerson C. (2017). Do electronic cigarettes increase cigarette smoking in UK adolescents? Evidence from a 12-month prospective study. Tob. Control.

[14] Hammond D., Reid J.L., Cole A.G., Leatherdale S.T. (2017). Electronic cigarette use and smoking initiation among youth: A longitudinal cohort study. Can. Med. Assoc. J..

[15] Soneji S., Barrington-Trimis J.L., Willis T.A., Leventhal A.M., Unger J.B., Gibson L.A., Ynag J. (2017). Associations between initial use of e-cigarettes and subsequent cigarette smoking among adolescents and young adults: A systematic review and meta-analysis. JAMA Pediatr..

[16] Bold K.W., Kong G., Camenga D.R., Simon P., Cavallo D.A., Morean M.E., Krishnan-Sarin S. (2017). Trajectories of E-Cigarette and Conventional Cigarette Use Among Youth. Pediatrics.

[17] East K., Hitchman S.C., Bakolis I., Williams S., Cheeseman H., Arnott D., McNeill A. (2018). The Association Between Smoking and Electronic Cigarette Use in a Cohort of Young People. J. Adolesc. Health.

[18] Schneider S., Diehl K. (2016). Vaping as a Catalyst for Smoking? An Initial Model on the Initiation of Electronic Cigarette Use and the Transition to Tobacco Smoking Among Adolescents. Nicotine Tob. Res..

[19] Leatherdale S.T., Brown K.S., Carson V., Childs R.A., Dubin J.A., Elliott S.J., Faulkner G., Hammond D., Mankse S., Sabiston C.M. (2014). The COMPASS study: A longitudinal hierarchical research platform for evaluating natural experiments related to changes in school-level programs, policies and built environment resources. BMC Public Health.

[20] Qian W., Battista K., Bredin C., Brown K.S., Leatherdale S.T. (2015). Assessing Longitudinal Data Linkage Results in the COMPASS Study.

[21] Wong S.L., Shields M., Leatherdale S., Malaison E., Hammond D. (2012). Assessment of validity of self-reported smoking status. Health Rep. Stat. Can..

[22] Tyas S.L., Pederson L.L. (1998). Psychosocial factors related to adolescent smoking: A critical review of the literature. Tob. Control.

[23] Barrington-Trimis J.L., Berhane K., Unger J.B., Cruz T.B., Huh J., Leventhal A.M., Urman R., Wang K., Howland S., Gilreath T.D. (2015). Psychosocial Factors Associated With Adolescent Electronic Cigarette and Cigarette Use. Pediatrics.

[24] Yuan M., Cross S.J., Loughlin S.E., Leslie F.M. (2015). Nicotine and the adolescent brain. J. Physiol..

[25] Counotte D.S., Smit A.B., Pattij T., Spijker S. (2011). Development of the motivational system during adolescence, and its sensitivity to disruption by nicotine. Dev. Cogn. Neurosci..

[26] Hammond D., White C.M., Czoli C.D., Martin C.L., Magennis P., Shiplo S. (2015). Retail availability and marketing of electronic cigarettes in Canada. Can. J. Public Health.

[27] Vanyukov M.M., Tarter R.E., Kirillova G.P., Kirisci L., Reynolds M.D., Kreek M.J., Conway K.P., Maher B.S., Iacono W.G., Bierit L. (2012). Common liability to addiction and “gateway hypothesis”: Theoretical, empirical and evolutionary perspective. Drug Alcohol Depend..

[28] DiFranza J.R. (2012). Which interventions against the sale of tobacco to minors can be expected to reduce smoking?. Tob. Control.

[29] Ontario Tobacco Research Unit (2018). The Tobacco and Vaping Products Act: Implications for E-cigarette Point-of-Sale Promotion. http://otru.org/wp-content/uploads/2018/09/update_sep.pdf.

[30] Smith R.F., McDonald C.G., Bergstrom H.C., Ehlinger D.G., Brielmaier J.M. (2015). Adolescent nicotine induces persisting changes in development of neural connectivity. Neurosci. Biobehav. Rev..

[31] Health Canada (2018). Tobacco and Vaping Products Act. https://www.canada.ca/en/health-canada/services/health-concerns/tobacco/legislation/federal-laws/tobacco-act.html.

[32] Lovato C., Watts A., Stead L.F. (2011). Impact of tobacco advertising and promotion on increasing adolescent smoking behaviours. Cochrane Database Syst. Rev..

